# Gender Effects in Observation of Robotic and Humanoid Actions

**DOI:** 10.3389/fpsyg.2020.00797

**Published:** 2020-04-30

**Authors:** Miriam Abel, Sinem Kuz, Harshal J. Patel, Henning Petruck, Christopher M. Schlick, Antonello Pellicano, Ferdinand C. Binkofski

**Affiliations:** ^1^Division for Clinical and Cognitive Sciences, Department of Neurology Medical Faculty, RWTH Aachen University, Aachen, Germany; ^2^Institute of Industrial Engineering and Ergonomics, RWTH Aachen University, Aachen, Germany; ^3^Institute of Neuroscience and Medicine (INM-4), Research Center Jülich GmbH, Jülich, Germany

**Keywords:** anthropomorphism, mirror neurons system, gender effect, human-robot interaction, motion perception, digital human model, gantry robot model

## Abstract

Robots are gaining an increasingly important role in industrial production. Notably, a high level of acceptance is an important factor for co-working situation between human and robot. The aim of the present study was to investigate the differences in the perception of anthropomorphic and robotic movements using models consisting of a virtual robot and a digital human. Videos of each model displayed different degrees of human likeness or robot likeness in speed and trajectories of placing movements. Female and male participants were asked to rate on a Likert scale the perceived levels of human likeness or robot likeness in the two models. Overall, results suggest that males were sensitive to the differences between robotic and anthropomorphic movements, whereas females showed no difference between them. However, compared to males, female participants attributed more anthropomorphic features to robotic movements. The study is a first step toward a more comprehensive understanding of the human ability to differentiate between anthropomorphic and robotic movements and suggests a crucial role of gender in the human-robot interaction.

## Introduction

Human-robot interaction is becoming prevalent in industrial production, healthcare industry, and rehabilitation (Karwowski, 1991). While safety is important in these contexts, human acceptance of robots as partners is essential for their successful implementation (Karwowski and Rahimi, 1991; Karwowski et al., 1988a,b, 1991a,b, 2018; Karwowski, 2019). In the same way, the advancement of comfort and trust is of primary importance in this progression (Lewis et al., 2018).

Currently, the influence of *anthropomorphism*, that is, the simulation of human characteristics by robots, is being investigated by several research groups (i.e., Duffy, 2003; Kuz et al., 2013). Conventional robots used in industry are fully automated systems that work reliably and efficiently without consideration for the human co-worker’s sense of comfort. Upon first glance humanoid robot systems seem to be more transparent and predictable than systems using traditional robots. Thus the worker should be able to trust the systems (Kuz et al., 2013) and should therefore exhibit an elevated rate of acceptance of the anthropomorphic robot as a co-worker. In line of principle, when this aim is reached, an optimal combination of human and anthropomorphic abilities is obtained that in turn brings to increases of productivity Krach et al., 2008).

Luczak et al. (2003), within a human-machine interaction context, investigated the effects of anthropomorphic design of technical devices. They found that humans perceive technical systems with anthropomorphic characteristic as being friendlier than simple devices. Hinds et al. (2004) have shown that humans exhibit increased trust with robots with an anthropomorphic appearance than with conventional robots. Furthermore, Krach et al. (2008) found a positive correlation between the anthropomorphic appearance and the attributed level of intelligence of the robot.

During interaction with robots human reactions are not only based on the robots’ appearance, but also on the motion path, the velocity, as well as the radius of their movements (Kuz et al., 2014). The way such motion parameters are arranged to render the movements of the robot more similar to human movements has also become a crucial factor for optimal human-machine interaction.

According to the *mental simulation theory* proposed by Jeannerod (2001) actual motor execution, and action observation are to some extent functionally equivalent as they are based on common motor representations. The brain mechanism able to match observed actions with the motor representation employed in the execution of those actions is known as the mirror neuron system (MNS) (Fabbri-Destro and Rizzolatti, 2008). Mirror Neurons have the peculiarity to discharge both when a given action is performed, and when the same action is observed as performed by someone else (Di Pellegrino et al., 1992; Gallese et al., 1996; Rizzolatti et al., 1996). This brought to the idea that movements which belong to our motor repertoire are recognized in someone else’s actions easier and faster than other movements which we do not typically perform (Kuz et al., 2013). Crucially, evidence of MNS activation has been provided also during the observation of robots: it has been observed that the more anthropomorphic features a robot movement had, the larger was the activation of MNS (Krach et al., 2008). Additionally, Gazzola et al. (2007) reported a similar activation of the MNS during observation of robot and human movements and suggested that the goal of an observed action is more important for mirror activations than the way the action is performed.

Other studies applied a human motion pattern to a gantry robot by tracking the human arm and elbow movements. The researchers found stress levels reduction in humans working in cooperation with a robot with tracked human-motion patterns (Zanchettin, 2012). These results are consistent with Huber et al. (2008) and Kuz et al. (2013) who found that anthropomorphic characteristics in gantry robots lead to reduced human reaction times, and suggested that humans are better in predicting the motion path and the endpoint of robot movements if they are anthropomorphic. In turn, such a better anticipation of movement endpoint makes worker feel safer, less stressed and more willing to work (Hugues et al., 2016). To summarize, anthropomorphic movements of a robot would activate the mirror neuron system of the observer; as a consequence, robot movements appear more natural and trustful and contribute to make the human-robot interaction as more sympathetic and pleasant.

One further point of interest in human-robot interaction is to understand whether gender is a crucial factor in the perception of anthropomorphic shapes of robots, as well as in the perception of anthropomorphic path movements against point-to-point robotic movements. As reported by the Statistics of the German Federal Labour Office (2019) the number of women in MINT professions has substantially increased in the last 26 years (Statistik der Bundesagentur für Arbeit, 2019). This brought to the important question of whether different settings should be applied for male and female workers to optimize interaction with robots and improve safety and productivity. Studies on emotional aspects of human-robot interaction have been conducted, which suggest that men would have more closeness to the robot as partner than female (Choi et al., 2017). However, to our knowledge, no systematic investigation of gender differences in human-robot interaction has been conducted yet, where robot anthropomorphism is manipulated through its appearance and its movements.

The aim of our study was to test the ability of two groups of male and female participants to differentiate between robotic and anthropomorphic movements performed by two different robot models: (1) a virtual representation of a gantry robot, and (2) a digital human model (see Figure 3).

We investigated:

(1) If the digital human model was perceived as more human than the gantry robot model, and if gender affected to some extent this difference.
(2) If the set anthropomorphic movement was perceived as more human than the robotic movement, and if gender affected to some extent this difference.
(3) Any possible interaction between gender, model, and movement that would ideally describe a continuum between a maximum and a minimum level of perceived anthropomorphism in the two gender groups, and eventually suggest any optimal human-robot setting for male and female users.

## Materials and Methods

### Participants

Forty right-handed healthy volunteers, twenty male, participated in the study (Table 1) after having given their written informed consent. They were recruited using the blackboard at RWTH University and University Hospital Aachen, mailing lists, and through word of mouth. They filled out a questionnaire to evaluate the inclusion criteria, which were: age (between 18 and 45 years old), right handedness, normal or corrected-to-normal vision, and absence of neurological or psychiatric diagnosis. Additionally, the career, the profession, and the highest degree of school education were recorded. There were no significant differences regarding age between the groups, *t*(38) = 1.12, *p* = 0.27. In the male group, 50% of the participants had technical occupations, whereas 70% of female participants had medical occupations. In both groups most of the participants had a high school degree at the point of the study. The groups did not differ significantly regarding occupation, *t*(38) = 1.25, *p* = 0.22 and school degree, *t*(38) = 0.23, *p* = 0.82 (for further information see Table 1). The study was approved by the Institutional Ethic Review Board of the Medical Faculty at RWTH Aachen University (EK 2013/14).

**TABLE 1:** Descriptive statistic of the participants.

		Male (*n* = 20)	Female (*n* = 20)
Age		24.8 (SD 5.9)	23.5 (SD 3.4)
Occupation	Medical	30%	70%
	Technical	50%	15%
	Business	10%	0%
	Others	10%	15%
School degree	High school	65%	55%
	University degree	30%	40%
	Professional training	0%	5%
	PhD	5%	0%

### Stimuli

**FIGURE 1:**
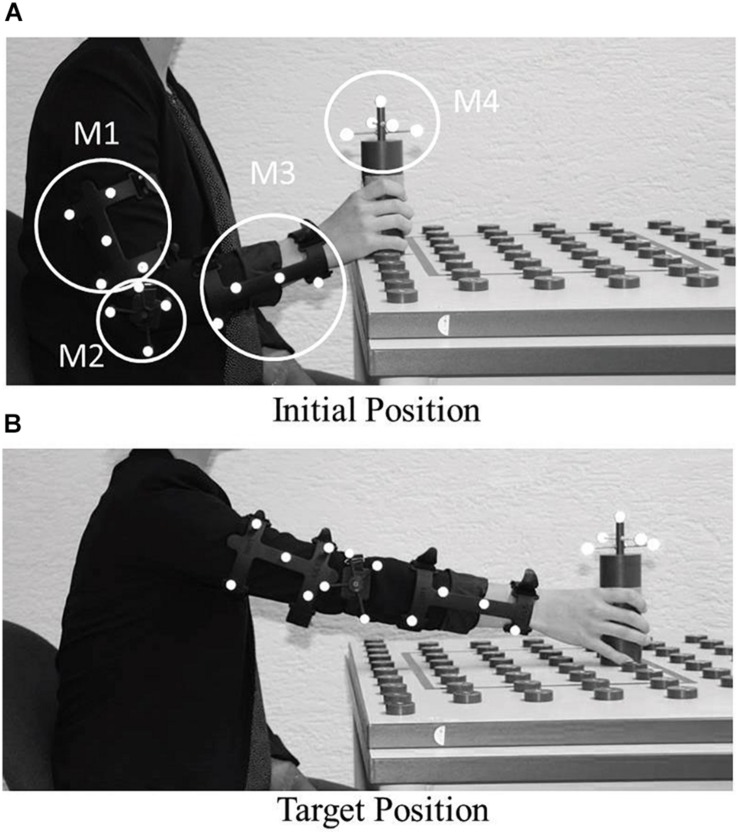
Experimental setting to track human placement movements (Kuz et al., 2015). **(A)** Initial position with the tracking markers M1, M2, M3, M4. **(B)** Target position.

**FIGURE 2:**
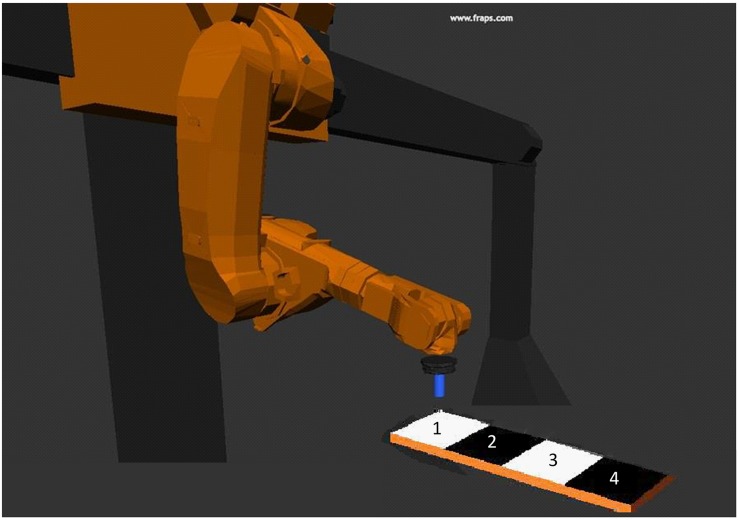
Example for robot model for the four movements on the grid pad we recorded for the human model and the robot model.

The video stimuli for two different models were generated with: (1) an Editor for Manual Work Activities (EMA) to simulate a human model (Fritzsche et al., 2011) and (2) a virtual gantry robot (Figure 2). The human motion data to be applied to the models were acquired from the right arm of a participant, using an optical 3D tracking system with four infrared cameras. Four markers were mounted on: the upper arm (M1), the elbow joint (M2), the forearm (M3) and on the cylinder (M4), respectively (Figure 1a). The participant was sitting in front of a table while holding a cylinder in the right hand and placing it on four predefined positions on the table (Figure 1). The arm movement had to start from the same position and with the same posture. For the analysis, the position of the markers in three-dimensional space, and the rotation of the markers were recorded. The arm was fully moved in the x,y plane, wherein the *y*-axis was directed upward. Only the rotation of the cylinder was considered in the three-dimensional space.

The collected data of the placing movements were adapted to both models: the digital human model and the virtual gantry robot model. As such, the joint angle of the shoulder, the elbow joint and the rotation of the hand were calculated. Finally, four different placing movements in the xy-plane were tracked. These movements were adapted to the models for the anthropomorphic movements. The point-to-point movements (robotic movements) were computed using the start and target positions from the tracked placing movements. The anthropomorphic movements followed a digressive (concave) curve whereas, the robotic movements followed a progressive (convex) curve. For further information about the generation of the motion data of the different videos please refer to Kuz et al. (2015).

Subsequently we programmed the virtual models of the human and the gantry robot using this generated motion data. Both models (human model and robot model) performed movements holding a cylinder in the tool center point (Figure 2). The points along the trajectory were arranged longitudinally in a row on a grid pad (Figure 2). Each movement from each model was counterbalanced relating to distance to the grid pad, joint angle for four different placing movements to the grid pad, and speed. In the final experiment, the grid pad was concealed. This was done not to indicate that the grid pad was precisely aimed for. All in all, we recorded eight movements for each model: position one, two, three, and four on the grid pad for human model and anthropomorphic movement; position one, two, three, and four on the grid pad for human model and robotic movement; the same eight records for the robot model (Figure 2). Position one has the closest distance to the model and position four has the largest distance.

For the final experiment we selected only three videos per model and movement. Indeed, we used videos of position two, three, and four, because the distance to position one was too close to observe differences between anthropomorphic and robotic movements. In the final experimental environment we presented twelve videos per block with a total of eight blocks. Each block had the same array: First, three anthropomorphic videos were presented with position two, three, and four, followed by three robotic movements with position two, three, and four. Subsequently, the same three anthropomorphic videos followed by the same three robotic videos were presented, with the same arrays.

### Study Design

**FIGURE 3:**
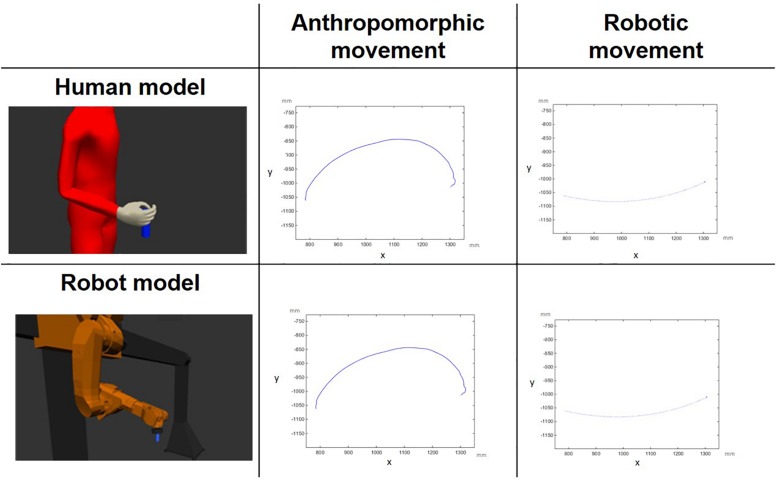
Visual representation of the factorial design: the depicted trajectories (projections on the X, Y plane) belong to a digital human model and to a gantry robot model performing anthropomorphic and robotic movements. The unit is millimeter (mm).

The study featured a factorial design with *gender* (male vs female) as the between-participants factor, and with *model* (human vs robot), and *movement type* (anthropomorphic vs robotic) as the within-participants factors. This resulted in four experimental conditions administered to each male and female group: human model performing anthropomorphic movement (HA), human model performing robotic movement (HR), robot model performing anthropomorphic movement (RA), and robot model performing robotic movement (RR, see Figure 3).

We instructed our participants to watch each video, and subsequently to judge the perceived level of anthropomorphism of the model movement in the video clip. The videos were presented in full color with a resolution of 900 × 563 pixels. The participant was allotted 10 s to rate each specific virtual model on a 5-point scale. Half of the participants used a scale from “very anthropomorphic” (score 1) to “very robotic” (score 5) and the other half from “very robotic” (score 1) to “very anthropomorphic” (score 5). To respond, the participants used a button box with three buttons (i.e., lateral buttons to go left and right on the scale and central button to confirm the response). Each participant completed eight blocks each with twelve videos (six anthropomorphic movement videos and six robotic movement videos). The conditions were counterbalanced across participants: half of the participants started the experiment with the human model (Block A) and half of the participants with the robot model (Block B). As described in 2.2 each participant has the same array of videos within a block. In total, each participant underwent four blocks each with twelve videos (six anthropomorphic movements, six robotic movements) of the human model and four blocks each with twelve videos (six anthropomorphic movements, six robotic movements) of the robot model.

### Analysis of Behavioral Data

The behavioral data analysis was based on subjective perception of movement assessed by using the 5-point scale. Responses from all participants were transferred to the same scale (5 = very anthropomorphic; 1 = very robotic). Data from male and female groups were tested for normality, *W*(20) = 0.97, *p* = 0.846 and *W*(20) = 0.95, *p* = 0.358, respectively; and for homogeneity of variance, *F*(1, 38) = 0.86, *p* = 0.360. Consistent with the planned model design, mean scores of the 5-point scale were submitted to an analysis of variance (ANOVA) with *gender* (male vs female) as the between-participants variable, and *model* (human vs robot) and *movement type* (anthropomorphic vs anthropomorphic) as within-participants variables. When necessary, paired and independent samples *t*-tests were performed as post-hoc comparisons with Bonferroni corrected *p*-value. An open-source tool was used to compute Cohen’s *d*_z_ effect size for the *t*-tests^1^.

## Results

**FIGURE 4:**
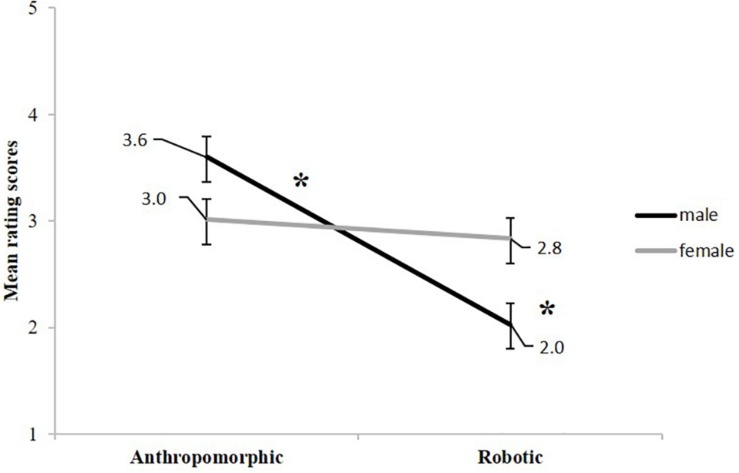
Gender x movement type interaction, including standard error bars. On the *y*-axis the 5-point rating scale is depicted (5 = very anthropomorphic; 1 = very robotic).

The main effect of gender was not significant, *F*(1, 38) < 1, *p* = 0.212, η^2^_p_ = 0.04 (male = 2.8; female = 2.9). Also the main effect of model was not significant, *F*(1, 38) < 0.001, indicating that the perception of a human (2.9) or of a robot model (2.9) had no impact on movement ratings. A significant main effect of movement type was observed, *F*(1, 38) = 9.103, *p* = 0.005, η^2^_p_ = 0.19 with anthropomorphic movements being rated significantly more anthropomorphic than robotic movements (3.3 vs 2.4). We found no significant interaction between model and movement type, *F*(1, 38) = 1.559, *p* = 0.219, η^2^_p_ = 0.04, and between gender and model, *F*(1, 38) = 0.439, *p* = 0.512, η^2^_p_ = 0.01. Nevertheless, the interaction of movement type and gender was significant *F*(1, 38) = 5.788, *p* = 0.021, η^2^_p_ = 0.13 (Figure 4). Paired samples *t*-tests displayed that male participants rated anthropomorphic movements more anthropomorphic than robotic movements, *t*(19) = 4.019, *p* < 0.001, *d*_z_ = 1.73; whereas female participants rated the two movements equally, *t*(19) = 0.414, *p* = 0.683, *d*_z_ = 0.18 (corrected *p* level = 0.025).

The possibility that female participants did not attend to the movements (thus reporting similar scores) was, in our opinion, highly improbable given the way the experiment was conducted. Indeed, female and male participants were tested in random order by the same experimenter who was present during the experiment.

Independent samples *t*-tests displayed that male participants rated the anthropomorphic movement as less anthropomorphic than female participants did, *t*(38) = 2.483, *p* = 0.018, *d*_z_ = 0.78 whereas their rating score of the anthropomorphic movement, despite being numerically higher, did not differ significantly from the female rating, *t*(38) = 2.114, *p* = 0.041, *d*_z_ = 0.67 (corrected *p* level = 0.025; Figure 4).

To verify whether the movement rating changed over time and interacted differently with the variables in the model, we computed for each participant the rating scores separately for the first and the second half of the experiment, and then submitted them to a follow-up 2 × 2 × 2 × 2 ANOVA with: gender × block (first vs second) × model × movement type.

We observed a main effect of block, *F*(1, 38) = 5.319, *p* = 0.027, η^2^_p_ = 0.02 displaying that rating scores increased slightly from the first to the second block (2.83 vs 2.90). Furthermore, block interacted with gender, *F*(1, 38) = 4.716, *p* = 0.036, η^2^_p_ = 0.11 showing that the scores increased slightly for the male group (2.73 vs 2.87), *t*(19) = 2.769, *p* = 0.012, *d*_z_ = 0.08, but not for the female group (2.92 vs 2.92), *t*(19) = 0.114, *p* = 0.910, *d*_z_ < 0.001. Crucially, block did not interact with any other variable showing that evaluation differences of perceived models-movements did not change from the first to the second half of the experiment in both the groups.

## Discussion

The aim of the study was to investigate whether human gender has a role in robot anthropomorphism within human-machine interaction contexts. A digital human model and a virtual gantry robot model performed both anthropomorphic path movements and point-to-point robotic movements. Two groups of male and female participants rated the perceived level of anthropomorphism of each of the four model and movement combination. Results clearly displayed that a human model was not perceived as more anthropomorphic than a robot model, whereas a movement mapped from human kinematics was perceived as more anthropomorphic than a standard robotic movement. Interestingly, male and female participants rated differently the two movement types. Overall, results suggest that males were sensitive to the differences between robotic and anthropomorphic movements, whereas females showed no difference between them. However, compared to males, female participants attributed more anthropomorphic features to robotic movements. Importantly, these differences were independent from the kind of “actor” that performed the movements, as no significant interaction was observed between movement type and model, and between gender, movement type and model.

Previous studies have highlighted gender differences in the execution of movements (Loovis and Butterfield, 1993; Roberton and Konczak, 2001; Junaid and Fellowes, 2009). In particular, studies that investigated the gross and fine motor skills from childhood through adolescence (e.g., Piek et al., 2006) suggest that gross motor skills are more salient in males relative to females behavior. The execution of both the anthropomorphic and the robotic movements implemented in the present study required gross motor skills; this could in part explain why male participants were more sensitive to the anthropomorphic/robotic features of these movements. Indeed following the MNS hypothesis, males’ motor system would be engaged in stronger motor simulations when perceived movements were anthropomorphic, as they would be part of participants’ own motor repertoire. In other terms, the higher rating for anthropomorphic movements requiring gross motor skills would be related to stronger motor simulations, whereas the lower rating for robotic movements would reflect weaker involvement of the MNS because they are less representative of males motor repertoire.

For their part, females’ lack of difference between the ratings of anthropomorphic and robotic movements depended on their anthropomorphic rating of robotic movement that was higher than the male group (together with the nonsignificant difference between the gender groups for the rating of anthropomorphic movements). Along the same MNS logic, this would mean that females’ MNS was more involved than males’ MNS with robotic movements, thus suggesting that motor simulation in females was less selective, that is, even movements that did not belong to their motor repertoire had more chances to “resonate” in their motor system. Similarly, female participants did not have weaker motor simulations when the perceived movements were anthropomorphic and required gross motor skills. Females’ MNS was still involved in motor simulations and contributed to a rating of anthropomorphic movements that was not statistically different from that of male participants. Thus, results support a link between the perceived level of anthropomorphism and the involvement of the MNS (Gazzola et al., 2007; Krach et al., 2008), and suggest a difference in sensitivity to anthropomorphism between males and females being related to different selectiveness of MNSs. To our knowledge, the latter opens a novel perspective that deserves further investigations.

For the sake of completeness, the present investigation did not collect information about individual skills and interests in the two groups that might have biased the group differences. For example, all the participants had no previous experience with industrial robots, but a more general familiarity with robots has not been controlled for, together with trust and anxiety toward robots. Notwithstanding these potential limitations, we think that our results essentially offer a reliable picture of gender differences in anthropomorphism.

Our results would have important implications in the design of human-robot interaction environments which could be personalized for male and female workers. To speculate, if in an assembly line, to perceive an anthropomorphic arm as moving like a human increases the ease and pleasantness of work, improves the productivity, and reduces harm risks (Oleson et al., 2011; Billings et al., 2012), then it would be more effective to implement human-like movements of machines when workers are males. Instead, as females tend to perceive robotic movements as more anthropomorphic than males do, implementation of anthropomorphic movements would be not as crucial.

Future research should employ imaging techniques to investigate gender differences in brain activation patterns associated to movements perception (anthropomorphic and robotic), as well as to the level of acceptance of anthropomorphic systems. This would allow insight into the underlying processing that mediate anthropomorphism in human-machine interaction, including the contribution of the MNS. This understanding could then be applied to the development and implementation of robots as co-workers in the manufacturing environment.

## Data Availability Statement

All datasets generated for this study are included in the article/supplementary material.

## Ethics Statement

The studies involving human participants were reviewed and approved by Ethikkommission an der Medizinischen Fakultät der Rheinisch-Westfälischen Technischen Hochschule Aachen. The patients/participants provided their written informed consent to participate in this study.

## Author Contributions

MA: planning and implementation of the study, evaluation and interpretation of the data, and writing the manuscript. SK and HP: planning and implementation of the study and evaluation of the video material. HP: implementation of the study and evaluation of the data. CS: supervising the study. AP: evaluation of the data and discussion of the results. FB: conception and supervision the study and discussion of the results.

## Conflict of Interest

The authors declare that the research was conducted in the absence of any commercial or financial relationships that could be construed as a potential conflict of interest.

## Footnotes

1 https://memory.psych.mun.ca/models/stats/effect_size.shtml
